# Editorial of the Special Issue: Cellular Mechanisms of Cardiovascular Disease

**DOI:** 10.3390/biomedicines11092494

**Published:** 2023-09-08

**Authors:** Tânia Martins-Marques, Gonçalo Coutinho, Attila Kiss

**Affiliations:** 1 Univ Coimbra, Coimbra Institute for Clinical and Biomedical Research (iCBR), Faculty of Medicine, 3000-548 Coimbra, Portugal; goncalofcoutinho@gmail.com; 2 Univ Coimbra, Center for Innovative Biomedicine and Biotechnology (CIBB), 3004-504 Coimbra, Portugal; 3 Clinical Academic Centre of Coimbra (CACC), 3004-561 Coimbra, Portugal; 4 University Hospital and Center of Coimbra, Cardiothoracic Surgery Department, 3000-075 Coimbra, Portugal; 5 Ludwig Boltzmann Institute for Cardiovascular Research, Center for Biomedical Research and Translational Surgery, Medical University of Vienna, 1090 Vienna, Austria; attila.kiss@meduniwien.ac.at

Cardiovascular diseases (CVD) remain the major cause of mortality and disability worldwide, having contributed to 19.1 million deaths in 2020. Although cardiovascular outcomes have significantly improved due to early diagnosis, improved and timely treatment, the prevalence of CVD is expected to increase in the coming years, namely due to population aging and other comorbidities highlighting the pressing need to identify novel biomarkers and disease-modifying treatments [1]. Therefore, a major future challenge in cardiovascular medicine lies in understanding the precise molecular basis of cardiac and vascular remodeling, which is the focus of this Special Issue.

Across the spectrum of CVD, numerous cellular and molecular changes have been reported to contribute to cardiac dysfunction [2]. In two review papers by Frąk et al., and Franczyk et al., the molecular mechanisms underlying the development and progression of CVD are comprehensively overviewed, focusing on the role of endothelial dysfunction, inflammation and oxidative stress as major drivers of atherosclerosis, coronary artery disease, hypertension and coronary artery spasm [3,4]. Both papers provide a relevant basis to identify potential diagnostic and therapeutic targets across different CVD.

Consistent with the impact of oxidative stress in CVD development, Islam et al. demonstrate that the H_2_S prodrug, SG-1002, confers protection against oxidative damage and hypertrophy in vitro [5]. At a mechanistic level, the authors suggest that the effects of SG-1002 are mediated by an increase in the H_2_S levels and expression of antioxidant proteins, with a concomitant decrease in the expression of atrial natriuretic peptide (ANP) and brain natriuretic peptide (BNP).

As the primary trigger for atherosclerosis, endothelial dysfunction has been a matter of intense research. In this Special Issue, Motoji et al. demonstrate that the administration of *Candida albicans* water-soluble fraction (CAWS) to an apolipoprotein-E-deficient (ApoE^−/−^) mice model induces a phenotype of Kawasaki disease (KD)-like vasculitis, exacerbating the formation of aortic plaque lesions [6]. Importantly, the authors show that administration of statins limited atherosclerosis and inflammatory cell infiltration, suggesting that statin therapy can be used to prevent cardiovascular events in KD patients.

Grounded on the important link between diet and vascular health, da Silva et al. investigated the impact of a ketogenic regimen in ApoE^−/−^ mice [7]. They demonstrated that a ketogenic diet promoted systemic inflammation and atherosclerotic plaque burden, which was accompanied by tissue-specific changes in aquaporins’ expression in the liver and adipose tissue. However, further studies are required to establish the link between nutritional ketosis and the regulatory role of aquaporins in inflammation during atherogenesis.

In addition to the mechanisms underlying endothelial dysfunction, it is important to have a significantly better knowledge of the pathways leading to the destabilization and rupture of atherosclerotic plaques that ultimately cause myocardial infarction (MI). In this Special Issue, Puylaert et al. identified Gasdermin D as a major player in pyroptotic cell death in atherosclerotic lesions [8], and demonstrated that Gasdermin D deficiency did not prevent plaque formation in the thoracic aorta of ApoE^−/−^ mice but delayed the progression of lesions in the brachiocephalic artery, with a concomitant decrease in plaque necrosis. Although additional evidence is required, this study suggests that Gasdermin D constitutes a valuable therapeutic target to increase atherosclerotic plaque stability.

The role of neutrophil extracellular traps (NETs) in atherothrombosis and plaque destabilization is thoroughly summarized in a systematic review by Nappi et al. [9]. The authors focus not only on the potential of using NETs as biomarkers of CVD, including acute coronary syndromes and peripheral arterial disease, but also on the therapeutic implications of targeting NETs in CVD.

Purinergic signaling plays a major role not only in regulating normal cardiovascular function, but also during pathological contexts, namely upon calcification of blood vessels and cardiac valves, which is the focus of the study carried out by Klauzen et al. [10]. In this paper, it is reported that valve interstitial cells (VIC) and valve endothelial cells (VEC) from stenotic human heart valves display significant changes in the expression levels of purinergic genes, which is associated with the pathological calcification of VIC, likely representing a valuable target in the search for an anti-calcification therapy.

The clinical benefits of using sodium–glucose cotransporter-2 inhibitors (SGLT-2i) have been shown and these stand a major therapeutic option for the treatment of heart failure (HF) independent of left ventricular ejection fraction (LVEF) and diabetes. However, the cardioprotective mechanisms of SGLT2i are still not fully known. In this Special Issue, Sullivan et al. focus on the importance of SGLT-2i in reducing edema in pre-clinical models of HF with reduced ejection fraction (HFrEF), highlighting the promising role of this strategy in improving the quality of life and reducing the number of hospitalizations of HFrEF patients [11].

It has been widely demonstrated that advanced glycation end-products (AGEs) contribute to a variety of microvascular and macrovascular complications. In this Special Issue, Heber et al. demonstrate that the levels of plasma methylglyoxal, a major AGE precursor, are increased in acute MI (AMI) patients following reperfusion via primary percutaneous coronary intervention [12]. Importantly, higher methylglyoxal levels within 24 h after AMI were associated with poorer left ventricular function after 4 days, suggesting that methylglyoxal constitutes a relevant and potential therapeutic target for post-MI remodeling and cardiac dysfunction.

In order to identify novel therapies for AMI, Docshin et al. characterized the mechanisms of early activation of regenerative processes in post-infarction cardiac tissue [13]. In this study, they demonstrated that cardiac mesenchymal cells isolated after MI in rats have an increased proliferative capacity, and a higher expression of the *Bmp2/Runx2* and Notch signaling genes, which have an important role upon cardiogenesis, paving the way for future studies aiming to ascertain its impact upon myocardial recovery in vivo.

Besides environmental and genetic causative factors, the epigenetic regulation of CVD-related genes is associated with the development and progression of multiple CVD [14]. In this Special Issue, Tarazón et al. observed a genome-wide hypomethylation in cardiac tissue samples of ischemic cardiomyopathy patients, which was related to changes in the DNMT3B system, the main DNA methyltransferase [15]. Overall, this study provides additional evidence to strengthen the potential of targeting epigenetic key enzymes for CVD treatment.

First described as an important epigenetic modification, by regulating chromatin structure and histone activity, protein acetylation is now known as a broader post-translational modification involved in the control of cell metabolism and enzymatic activity. In this Special Issue, Dubois-Deruy et al. discuss the role of lysine acetyltransferases and deacetylases in the pathophysiology of obesity, type 2 diabetes and HF, as well as the preclinical evidence demonstrating the cardioprotective potential of the pharmacological modulation of cardiac acetylation [16].

In addition to classical risk factors, CVD burden is now recognized to be aggravated due to the growing population of cancer survivors treated with cardiotoxic cancer therapies, which is discussed in two review papers collected in this Special Issue. Franczyk et al. consider evidence relating the use of receptor tyrosine kinase inhibitors (RTKIs) in treatment of renal cell carcinoma to the development of hypertension, myocardial ischemia and HF, highlighting the importance of defining proper surveillance strategies for high-risk patients [17]. Rocca et al. focus on mitochondrial dynamics as key determinants of anticancer drug-dependent cardiotoxicity, which constitute relevant therapeutic targets to prevent cardiomyocyte dysfunction or loss in cancer survivors [18].

Besides anticancer drugs, therapeutic regimens used for systemic disorders have also been reported to increase HF-associated hospitalizations, such as the dipeptidyl-peptidase-4 (DPP4) inhibitor saxagliptin, widely used to control type 2 diabetes. In the paper by Vörös et al., the mechanisms underlying saxagliptin cardiotoxicity are investigated [19]. The authors demonstrate that the levels of DPP4 and its substrate neuropeptide Y (NPY) are decreased in failing human hearts. In vitro experiments using fibroblast/cardiomyocyte co-cultures suggest that saxagliptin may interfere with NPY-mediated cardiac tissue remodeling in HF, but further studies are warranted to confirm this association.

Overall, this Special Issue brings together relevant cell-based and pre-clinical studies identifying novel mechanisms underlying cardiac and vascular remodeling and dysfunction, paving the way for the identification of more efficient diagnostic and therapeutic tools for a wide range of CVD.
